# Food “Vitamines”

**Published:** 1913-11-15

**Authors:** 


### FOOD "VITAMINES."

Dr. Leonard Hill said he had come over from the Section of Physiology to say a few words on food values. It was now clearly established that the vital qualities of nature's foods, called "vitamines," were destroyed by the modern methods of sterilization and milling. White bread, for instance, contained none of these "vitamines." The effect of eating white bread did not matter to the classes who could afford to buy milk, eggs, meat and vegetables. But the consequences were serious to the health and vitality of the working classes, who were able to supplement their white bread only with jam and tea, which were also lacking in "vitamines." Some years ago the political world was greatly disturbed on the question whether the British working classes, like the German, were to be reduced to the eating of "black bread." At that time very little was known about "vitamines," otherwise black bread would have carried the day. It was possible to sustain life on black bread and water, but not on white bread and water.

FOOD OF ULSTER FACTORY GIRLS.

During a recent visit to Ulster he inquired into the diet of the girls employed in the flax spinning sheds. A hot moist atmosphere was necessary in the sheds for the purposes of the spinning. Such an atmosphere made a great strain upon the physical energies of the spinners, and it was necessary for the maintenance of their health and vitality that they should eat the most wholesome food. In one district there was a model lodging-house in which a large number of those girls lived, paying 5s. a week, and the following were their daily meals:

On rising at 5.45 o'clock.—Tea and white bread.

Breakfast, 8 o'clock.—Tea and white bread with egg or bacon.

Dinner, 1 o'clock.—Broth and bacon, tea and white bread.

Tea, 5.45 o'clock.—Tea, white bread and jam and margarine.

Supper, 10 o'clock.—Tea, white bread and jam.

The girls therefore had a great many meals, but those meals contained very little nutrition. Semi-starvation could be produced by wrong feeding as well as by insufficient feeding. The tea these girls drank—black tea, brewed in a pot—was also most injurious to the nerves. The jam was also to be condemned. Sugar was but an artificial foodstuff. A little sugar was an excellent thing, but to make it a food, as many young people did by consuming large quantities of it in the form of sweets, was a most mischievous practice.

Professor Meredith, Queen's University, Belfast, asked whether the views so dogmatically expressed by Dr. Hill were authoritative and final, or were simply his own personal opinions.

Dr. Hill said the views he had expressed were the result of experiments carried out by competent men in almost every country in the world.

Mr. C. R. Fay, Christ's College, Cambridge, asked whether they were to conclude that the recent movement in favor of brown bread was a healthy movement.

Dr. Hill replied that so far as it affected the poorer classes it was a very healthy movement.

Miss Matheson, Birmingham Women's Settlement, said that in the consideration of the question the great influence of tradition upon the working classes must not be lost sight of. If a commodity that was a favorite article of consumption with the working classes became dearer, the average working man would still go on paying the dearer price as long as he possibly could rather than change. In fact, it took years to bring about the substitution of one article of consumption for another in working-class families. That was shown during the coal strike. Women whose husbands were earning only 18s. a week cheerfully paid two or three shillings per cwt. for coal which they got for 10d. in normal times, rather than adopt a cheap system of cooking that was brought to their notice by the Women's Settlement. Thus tradition changed much more slowly than price.—*Dental Record.*

